# The Evolution of Antisense Oligonucleotide Chemistry—A Personal Journey

**DOI:** 10.3390/biomedicines9050503

**Published:** 2021-05-03

**Authors:** Sudhir Agrawal

**Affiliations:** 1 ARNAY Sciences LLC, Shrewsbury, MA 01545, USA; Sagrawal@arnaysciences.com or Sudhir.Agrawal@umassmed.edu; 2 Department of Medicine, University of Massachusetts Medical School, 55 N Lake Ave, Worcester, MA 01655, USA

**Keywords:** oligonucleotide, therapeutic, antisense, medicinal chemistry, structure activity relationship

## Abstract

Over the last four decades, tremendous progress has been made in use of synthetic oligonucleotides as therapeutics. This has been possible largely by introducing chemical modifications to provide drug like properties to oligonucleotides. In this article I have summarized twists and turns on use of chemical modifications and their road to success and highlight areas of future directions.

## 1. Introduction

Today RNA therapeutics are a clinical reality with approved drugs that employ a variety of different mechanisms of action (reviewed recently in [1]). However, here I would like to take you back to the beginnings of antisense and show you how the evolution of antisense chemistry played an essential role in turning oligonucleotides into therapeutics. I would like to dedicate this chapter to Paul Zamecnik (1912–2009), my mentor, friend, and colleague who introduced me to the field of antisense therapeutics and who first dreamed of using antisense oligonucleotides as therapeutics [2]. To achieve his dream, we had to improve antisense oligonucleotide chemistry, understand how the chemistry, structure, and sequence influences antisense oligonucleotide function, and how the immune system deals with exogenous nucleic acids. All this is research I was and still am personally involved in, so I will mainly focus on Paul’s and my own thinking and how it changed over time as we experimented and accumulated data.

## 2. The Beginning

But first, let me tell you how I came to work with Paul. Starting in 1985, I was conducting post-doctoral research on the synthetic chemistry of nucleic acids with Michael Gait at the Laboratory of Molecular Biology (LMB), Medical Research Counsel, in Cambridge, UK [3]. During this period, one of the scientists I was very close to was the late Dr. Dan Brown (1923–2012) [4]. Dan was an astounding nucleotide chemist, who, together with future Nobel Laureate Alexander Todd, had determined the nature of the chemical linkages in DNA and RNA in 1951. In 1953, whilst working in Todd’s lab, Dan met Paul Zamecnik, who had an interest in protein synthesis and was visiting Todd’s lab. At that point, their interests did not overlap much, so it remained a brief meeting.

However, later in his career, Paul’s interest turned to using nucleic acids as therapeutics. In 1978, Paul published two papers showing that a synthetic oligonucleotide complimentary to the mRNA sequence of Rous Sarcoma virus could bind to that mRNA and thereby inhibit viral replication [5,6]. Paul referred to such oligonucleotides as “hybridons” though later these became known as antisense oligonucleotides. Further work in this area was very slow as methodologies to synthesize oligonucleotides and sequence DNA were not available yet—Sanger sequencing was only published in 1977 and not commercially available until 1986. However, Paul persisted in his research and during the early 1980s applied the antisense approach to the newly identified human retrovirus HIV-1, findings he published in 1986 [7] and that were later corroborated by Samuel Broder’s lab [8].

Seeking advice on the chemistry of oligonucleotides, Paul reconnected with Dan, and during one fateful call Dan mentioned my work on nucleic acid chemistry to Paul. The next day, I received a call from Paul and, after a brief conversation, he invited me to join his laboratory at the Worcester Foundation for Experimental Biology, Shrewsbury, MA to pursue work on antisense chemistry. 

## 3. Stability against Nucleolytic Degradation

**Figure 1:**
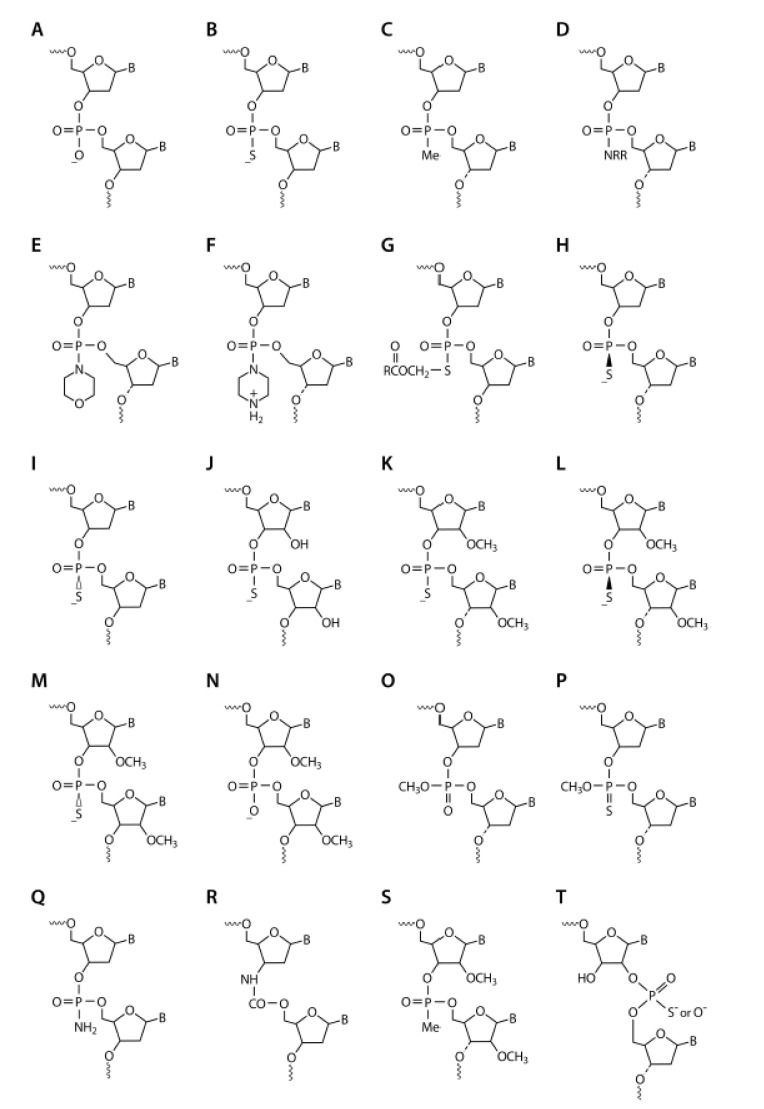
Chemical structures: (**A**) phosphodiester oligonucleotide (PO-ODN), (**B**) phosphorothioate ODN (PS-ODN), (**C**) methylphosphonate ODN (PME-ODN), (**D**) phosphorodiamidate, (**E**) phosphomorpholidate,(**F**) phosphopiperazidate (PN-ODN), (**G**) acyloxyester PS-ODN, (**H**) *R*p-PS-ODN, (**I**) *S*p-PS-ODN, (**J**) PS-oligoribonucleotide (PS-ORN), (**K**) 2′-O-methyl PS-ORN (2′PS-ORN), (**L**) *Rp* 2′ PS-ORN, (**M**) *Sp* 2′ PS-ORN, (**N**), 2′ PO-ORN, (**O**) methylphosphotriester, (**P**) methylphosphothiotriester, (**Q**) primary phosphoramidate, (**R**) carbamate, (**S**) 2′-O-methylphosphonate, and (**T**) 2′-5′-ribonucleoside; B = heterocyclic base.

When I started in Paul’s lab in 1987, our overall goal was to provide drug-like properties to the nucleotide sequence of antisense oligonucleotides so that they could be used as therapeutics. Our first objective was to provide nuclease stability to the antisense oligonucleotides so that they would survive longer in biological fluids. At the time, it was hypothesized that the polyanionic internucleotide linkages would interfere with cellular uptake, so we focused on non-ionic modifications. We decided to pursue three particular internucleotide chemical modifications: phosphorothioate (PS; Figure 1B), methylphosphonate (P-ME; Figure 1C), and phosphoramidate (P-N; Figure 1D). 

PS-ODNs had been extensively studied by Eckstein and colleagues in the 1970s and early 1980s [9]. The first semi-automated oligonucleotide synthesizers had been developed in 1982, and I had used an early version of these to synthesize oligonucleotides in Mike Gait’s lab in the mid-19080s. These synthesizers became fully automated by 1988. To obtain large quantities of PS-oligodeoxynucleotides (PS-ODNs), we had to optimize PS synthetic methodology and adapt it for use in these automated synthesizers. Also in the early 1980s, Paul Tso and Paul Miller had published a series of papers on P-ME-ODNs and their characteristics [10]. In these P-ME-ODNs, one of the oxygens in the phosphate backbone linkage is replaced with a methyl group, analogous to sulfur replacing the oxygen in PS-ODN. However, unlike the sulfur replacement, the methyl group also removes the negative charge, leaving the linkage non-ionic. Tso and Miller had manually synthesized short P-ME-ODNs using activated nucleoside methylphosphonates, but this method did not lend itself to the production of the larger quantities and longer P-ME-ODNs that we needed, so we pioneered the use of nucleoside methylphosphonamidites as building blocks in automated synthesizers [11]. We also synthesized various antisense ODNs containing P-N- internucleotide linkages with different ionic states, including phosphomorpholidate, N-butyl phosphoramidate, and phosphopiperazidate (Figure 1D–F, respectively). 

All of these antisense oligonucleotides were 15–20 bases long and targeted to HIV-1 [12,13]. The nuclease stability of P-ME- and P-N-ODNs was clearly higher than that of PS-ODNs, which itself was significantly more stable than phosphodiester (P-O) ODNs (Figure 1A). To evaluate affinity to the target mRNA we recorded melting temperatures when bound to complimentary RNA; all of the modified antisense ODNs demonstrated lower affinities than the P-O-ODNs [13]. When testing the potential for HIV inhibition in cells, PS-ODNs were consistently more potent than any of the other modifications [13,14,15]. P-ME- and P-N-ODN also had issues with solubility in biological media, so we quickly focused on PS-ODNs. Having observed the potential of antisense PS-ODNs as anti-HIV agents, we expanded our studies to include activity against the influenza virus and confirmed that such ODNs could also inhibit influenza virus replication [16]. 

Further insights into the observed increased potency of PS-ODN antisense came from studies which showed that when PS-ODNs was hybridized with target RNA, RNase-H was activated and subsequently cut the targeted RNA at the duplex site [17]. This mechanism allows one PS-ODN antisense molecule to cleave multiple RNA strands, making PS-ODN antisense catalytic. P-ME- and P-N-ODNs on the other hand were unable to activate RNase-H [17]. However, the efficacy of PS-antisense ODNs in activating RNase-H was somewhat lower than that of P-O-ODNs, suggesting that the PS modification affected RNase-H recruitment. At the time, we theorized that this was perhaps due to differing affinity and/or presence of stereoisomers due to the chiral center of the PS modification. 

These early studies with PS-, P-ME-, and various P-N-antisense ODNs provided us with key insights into which characteristics of an ODN are important for both antisense potency and mechanism of action. We came to understand the following points: Firstly, PS- ODNs bind to target mRNAs and these DNA/RNA hybrids are substrates for RNase-H, resulting in cleavage of the target mRNA and thus reduced protein expression. Secondly, P-ME-ODN and P-N-antisense ODNs can bind to target RNA and inhibit translation by steric hindrance. Thirdly, the RNase-H-based mechanism of action is more potent than the steric hindrance mechanism because of its catalytic nature. Lastly, we realized that the differing properties of chemical modifications could be combined to modulate ODN stability characteristics but at the same time retain RNase-H activation. We referred to this new generation of ODNs as mixed-backbone antisense [18]. 

As a follow-up to these publications, an increasing number of papers started to appear in which PS-antisense ODNs were employed against various RNA targets. PS-ODN antisense became the modification of choice for first generation antisense and several biotechnology companies were founded to develop such antisense therapeutics. Among them was Hybridon, later called Idera Pharmaceuticals, founded by Paul, and which I joined as a founding scientist.

## 4. PS-ODNS—First Generation Antisense Therapeutics

At Hybridon, with what we thought where functional ODNs in hand, we set out to investigate in-vivo delivery of PS-ODNs. We carried out the first study in mice by administering S^35^-labeled PS-ODNs, both intravenously and subcutaneously, and evaluated the disposition, excretion, and metabolism [19]. We found that PS-ODNs had a short plasma half-life, tissue disposition was broad, and the primary route of elimination was urinary excretion. In contrast to PS-ODNs, P-ME-ODNs had a very short plasma half-life and over 70% of the administered dose was excreted in the urine within 2 h. This discrepancy suggested to us that PS-ODNs were likely binding to plasma proteins and we thought that that property might be critical for their retention and disposition [20]. Extensive work on this subject has been done by the Crooke lab [21]. Further support of this observation came from our studies in which the administration of aspirin, which modulates serum protein binding, affected the disposition of PS-ODNs [22]. Analysis of PS-ODNs by PAGE showed time-dependent degradation and intact PS-ODN was detected for several days in multiple tissues, including the liver and kidney. PS-ODN did not cross the blood–brain barrier as there was very little disposition in brain tissues. The degradation of PS-ODNs was primarily from the 3′- end as capping of the 3′- end significantly reduced degradation in vivo [23]. Pharmacokinetic studies of PS-ODNs in rats [24], non-human primates [25], and humans [26] showed very similar profiles to what we saw in mice. 

The next step for us was to learn about the safety of PS-ODN in mice, rats, and non-human primates. Subcutaneous administration of PS-ODN in mice and rats showed dose-dependent safety signals including changes in the clinical chemistry and histology of multiple organs [27,28,29]. These histological changes appeared to be largely due to inflammation. Bolus intravenous administration in non-human primates led to significant hemodynamic changes [30]. Extensive work in our laboratory led to the discovery that these changes were associated with activation of the complement system (for example, CH50, C5a). The changes were independent of PS-ODN sequence but dependent on the dose and rate of infusion, and reducing the rate of intravenous infusion or using subcutaneous administration mitigated these hemodynamic changes. Based on our publication, FDA staff published guidelines on administration of PS-ODNs and recommended the inclusion of non-human primates as one of the species in non-clinical safety studies of PS-ODN antisense [31]. 

We soon realized that for clinical development of PS-antisense ODNs we would need a large amount of material manufactured under GMP conditions. So we went back and optimized the synthetic methodologies and purification of PS-ODNs for scales up to 5 mmol which is equivalent to multiple grams of PS-ODN [32]. We also set up the first manufacturing unit, HSP in Milford, MA, now owned and operated by Avecia [33]. 

Thinking that we had addressed all possible issues, we initiated a clinical trial in HIV-infected individuals with our lead candidate, GEM91, in 1993. In this trial, GEM91 was administered by intravenous (IV) infusion over 8 days at dose-levels of 3.2 mg/kg/day to 4.4 mg/kg/day, or by subcutaneous administration. We observed dose dependent injection site reactions, flu like symptoms, and thrombocytopenia in a few individuals. These effects were more pronounced with subcutaneous (SC) administration than IV-infusion and individuals treated with SC administration had transient swelling of the draining lymph nodes. Plasma HIV-1 load remained largely unchanged in the placebo group, but curiously, in treated subjects there were dose dependent temporary increases in HIV-1 load from day 4 to 6. By day 8, HIV levels in treated subjects returned to similar levels as those in the placebo group [34]. Further dosing in the trials was discontinued due to this observation. 

## 5. Modified PS-Oligoribonucleotides (PS-ORNs)

In parallel to studying PS-ODNs, we also investigated PS-oligoribonucleotides (PS-ORNs) and 2′ substituted PS-ORNs (2′PS-ORNs) as antisense agents. PS-ORNs and 2′PS -ORNs showed increased affinity to complementary RNA compared to PS-ODNs and similar or increased nuclease stability, but did not activate RNase-H. However, in comparison to PS-ODNs, PS-ORN and 2′PS-ORNs showed reduced potency in HIV-1 targeting studies [35,36]. We already knew that RNA/RNA hybrids were not a preferred substrate for RNase-H, so this result was a further confirmation that activation of RNase-H was critical for effective RNA knockdown. 

However, we thought that such PS- and 2′PS-ORN might still be useful in antisense oligonucleotides intended to sterically block and thus modulate RNA processing mechanisms, for example alternative splicing. An early indication that this could work came from studies in which we employed P-ME antisense to mask U1 and U2 snRNPs that are required for spliceosome assembly [37]. To confirm the utility of these oligonucleotides, we collaborated with Ryszard Kole. We treated mammalian cells expressing an incorrectly spliced human betaglobin gene with 2′PS-ORNs targeted at the aberrant splice sites. This restored the normal splicing pattern and generated the correct human betaglobin mRNA and polypeptide [38]. In collaboration with Ryszard Kole and Steve Wilton, we later extended this observation to dystrophin, an often mis-spliced protein that can cause Duchenne Muscular Dystrophy [39]. 

While we were pursuing these functional studies, we were also accumulating data on other characteristics of PS- and 2′PS-ORNs. We observed that 2′PS-modified ORNs had minimal effect on complement activation and prolongation of aPTT and also had reduced inflammatory responses [40]. 

## 6. Putting the Pieces Together: Mixed Backbone Antisense

It became clear that PS-ODNs had certain desirable characteristics such as good nuclease stability, target affinity, RNase-H activation, in-vivo delivery, and tissue disposition. However, they also displayed a few undesirable characteristics such as their polyanionic nature, activation of the complement system, aPTT prolongation, and sequence dependent immune activation. Similarly, 2′ PS-ORNs had desirable characteristics, for example increased nuclease stability and affinity to the target RNA as well as reduced polyanionic nature, complement activation, and inflammatory responses. However, their big drawback was the lack of RNase-H activation. 

**Figure 2:**
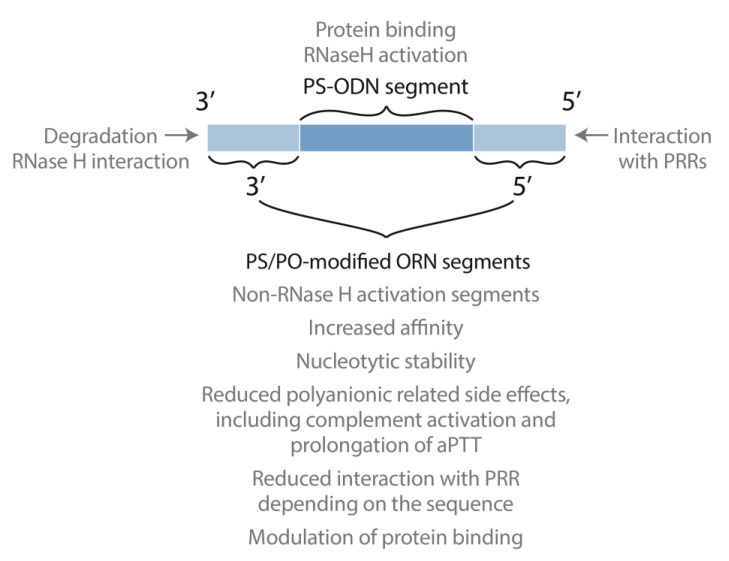
Design of mixed backbone antisense oligonucleotides.

We thought that perhaps we could combine the desirable properties of PS-ODN and 2′PS-ORN into one antisense molecule by combining segments containing the different modifications and nucleic acid types into one ODN (Figure 2). We referred to this type of antisense as mixed-backbone oligonucleotides or hybrid, now also called gapmers [35,36]. To elucidate the ideal combination of segments, we studied versions with varied positioning of the PS-DNA and 2′ PS-ORN segments. In one configuration, we placed PS-DNA in the center of the antisense molecule, flanked by 2′PS-ORN at both the 3′- and 5′- end (Figure 2) [41,42]. Conversely, we placed the 2′PS-ORN in the middle and segments of PS-ODN at both the 3′- and 5′- end. Both configurations showed promising results in various studies compared to pure PS-ODNs. However, gapmers with the PS-DNA segment in the center became the preferred choice and have since been extensively pursued by investigators around the world.

In a mixed backbone antisense, segments of PS-DNA and modified RNA (or otherwise non-RNase H activating) are appropriately placed to combine the desirable characteristics for these different modifications into one antisense agent. The PS-DNA segment allows for RNase H activation and protein binding to allow tissue distribution while the modified RNA (or otherwise non-RNase H activating) segments provide increased nucleolytic stability, affinity to target RNA, decreased polyanionic characteristics and reduced inflammatory responses. Modifications at the 5′- end modulate interactions with PRRs, and modifications at the 3′- end increase nucleolytic stability.

Compared to the PS-ODN first generation design, gapmers have shown increased potency against many gene targets in cell culture and in-vivo disease models. They have demonstrated improved safety in rodents and primates [27] and reduced polyanionic related side effects including complement activation and prolonged activation of aPTT [40]. Pharmacokinetic studies of gapmer antisense in mice showed similar tissue disposition as PS-ODNs, but significant increases in stability in multiple tissues, making less frequent dosing possible [43]. The increased in-vivo stability allowed us to study oral and colorectal delivery [44], for example, an orally administered gapmer has shown anti-tumor activity in a murine tumor model [45]. 

Additionally, we realized that gapmers containing segments of PS-ODN and 2′PS- ORN were very resistant to degradation in vivo. This allowed us to use PO linkages in the 2′PS-ORN segments to further modulate polyanionic-related side effects and protein binding [46]. The drug-like characteristics of gapmers could also be enhanced by strategically incorporating certain internucleotide linkages, such as P-ME (Figure 1C) [17], P-N (Figure 1F) [17], methylphosphotriester (Figure 1O) [47,48], primary phosphoramidates (Figure 1Q) [49], carbamates (Figure 1R) [50], 2′-O-methylphosphonates (Figure 1S) [51], and 2′-5′-ribonucleosides (Figure 1T) [52].

In 2001, Isis Pharmaceuticals (now Ionis Pharmaceuticals) took a license of the gapmer antisense chemistry and other associated patents from us [53]. Currently, most of the RNase-H-mediated antisense drug candidates in development employ the gapmer antisense design. Over the years, many different 2′-O-modifications have been studied but currently 2′-O-methyl (OME; Figure 1K) and 2′-O-mythoxyethoxy (MOE) modifications remain the standard [54]. These have been discussed in detail recently [54,55]. 

## 7. Paying the Tolls: The Role of Sequence and Modifications

From our studies of PS-ODNs it became evident that PS-ODNs activate immune responses in a sequence-dependent manner [56,57]. We observed very clear evidence of this in mouse models where antisense ODNs targeting human Papilloma Virus also inhibited other viral infections. Yet, anti-viral activity was abolished when the same experiments were done in immune-compromised mice [58]. We were baffled by what was happening and then the human studies with GEM91 actually showed a temporary increase in HIV load [59]. We certainly did not expect that and were keen to understand the underlying mechanisms. 

By the late 90s, there had been a few reports of the immune-stimulatory properties of nucleic acids, but few details were known [60,61,62,63,64]. Further insights came with the confirmation that previously only hypothesized pattern recognition receptors (PRRs) existed in 1997. PRRs are a family of proteins expressed on various immune cells that recognize pathogen-associated molecular patterns (PAMPs) of microbes and subsequently initiate downstream innate immune responses to protect the host from infection. 

**Figure 3:**
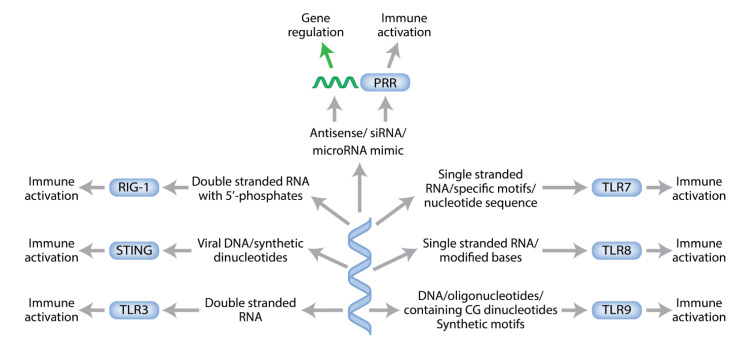
Pattern recognition receptors (PRRs).

Since that initial discovery, it has become clear that there are a large number of such PRRs and that some of these recognize specific PAMPs present in endogenous or exogenous nucleic acids. Pattern recognition receptors include Toll-like receptors (TLRs), RIG-like receptors (RIG-I), and inflammasomes [65]. Of specific interest for nucleic acid therapeutics are TLR-3 (recognizes double-stranded RNA), TLR-7 (single-stranded RNA), TLR-8 (single stranded RNA containing modified bases), and TLR-9 (DNA containing unmethylated CG dinucleotides; Figure 3). Inflammasomes are activated by double-stranded DNA, and RIG-I recognizes RNA with 5′ phosphates. 

These PRRs are known to recognize nucleic acids and induce immune responses. Each receptor is expressed on defined cells and recognizes specific pathogen-associated molecular patterns (PAMPs) of nucleic acids. Particular types of immune responses are induced depending on the exact nucleotide sequence and secondary structure of the nucleic acids acting as PAMPs.

Up until the discovery of these PRRs, work on chemical modifications of antisense focused on improving affinity, nuclease stability, RNase-H activation, and delivery and mitigation of polyanionic characteristics. But the discovery of PRRs together with our perplexing results from the GEM91 human trials and the mouse viral infection studies, led us to investigate the immune modulatory activity of therapeutic oligonucleotides. Specifically, we were interested in the role of nucleotide sequence, nucleotide motifs, and chemical modifications in immune activation. For initial studies we chose a representative PS-ODN containing a CG dinucleotide in the center [66]. This sequence resulted in TLR-9-mediated immune responses and induced secretion of cytokines including IL-12 and IL-6. We substituted each nucleotide in the flanking sequence at both the 5′- and 3′- ends with 2′-O-methylnucleoside, P-ME internucleotide linkages, or with a basic nucleoside. These substitutions had a significant effect on immune-stimulatory activity but only in defined positions [66]. Substitutions made in the 5′- flanking region had notable effects—if these 5′ substitutions were close to CG dinucleotides, they significantly mitigated the immune-stimulatory activity of the CG. Substitutions four or more bases distant from the CG had little effect but changed the ratio of IL-12 and IL-6 while substitutions in the 3′- end had little to no effect [67,68]. 

This indicated to us that the 5′- end of PS-ODN has different characteristics than the 3′- end [69,70,71]. To investigate this further, we blocked access to the 5′- end either by linking two PS-ODNs via 5′-5′ linkages or by conjugation with a bulky ligand [72]. We saw significant reductions in immune-stimulatory activity, suggesting that the accessibility of the 5′- end is key for the immune activity of PS-ODNs [71]. At the same time, linking two PS-ODNs via 3′-3′ linkages significantly increased immune-stimulatory activity [73]. If PS-ODNs contained CG dinucleotides towards both the 5′ and 3′ ends, modification of the 5′ CG mitigated immune-stimulatory activity much more than modification of the 3′ CG-dinucleotide [74]. We also evaluated if nucleoside modifications would have an impact on immune-stimulatory activity. Replacing the C or G in the CG dinucleotide with a number of different nucleosides failed to activate immune responses (5-methylcytosine, 5-methylisocytosine, 5-hydroxycytosine, uracil or inosine, 2′ -aminopurine, nebularine, 8-oxoguanosine, and iso-guanosine, respectively) [75,76,77]. Interestingly, incorporation of 5-methylcytosine in CG dinucleotides makes PS-ODNs act as TLR9 antagonists and is now routinely employed as a substitute of C in current antisense oligonucleotides. Incorporation of N4-ethylcytosine or 7-deaza guanosine still activated immune responses [75,76]. These studies provided us with a clear indication that nucleosides could alter the interaction with PRRs depending on the sequence context. 

PS-ORNs and analogs had been shown to interact with TLR-3, TLR-7, and TLR-8. Since these segments are part of the gapmer design, we conducted detailed structure activity relationship studies to further characterize these compounds. Results from these studies have been reported by us in a series of papers [78,79,80]. In summary, activation of TLR-7 or TLR-8 leads to different immune response profiles, and the profiles are dependent on the nucleoside composition, presence of modified nucleosides, accessibility of the 5′- and 3′- ends and secondary structure. The presence of secondary structures with palindromic regions will also activate TLR-3 [81]. 

### Immunotherapy with Synthetic Oligonucleotides

While our original goal was to understand the interaction of PS-ODNs with PRRs so that we could design better antisense oligonucleotides, we ended up with great insights into how to design an optimal TLR9 agonist instead. We now had a library of synthetic immune-stimulatory dinucleotide motifs, and an understanding of the need for the accessibility of the 5′ end and how length, nucleotide composition, and secondary structures resulted in different types of immune responses. So, in 2007 we changed research directions and to reflect that change, we changed our company name from Hybridon to Idera Pharmaceuticals. We created a library of novel TLR9 agonists, and studied their therapeutic utility as vaccine adjuvants [82,83,84] and anti-viral agents [85], in the treatment of asthma and allergies [86], and in immunotherapy for cancers [87,88,89,90]. Currently, a TLR-9 agonist, IMO-2125, is in clinical trials for immunotherapy for multiple tumor types and has shown promising results in melanoma patients [91]. 

Similar studies of PS-ORN have guided us in how to avoid interactions with PRRs for antisense oligonucleotides. At the same time, the studies also taught us how to create optimal agonists of TLR-7 and TLR-8, or dual agonists of TLR-7 and 8, and agonists of TLR-3. Such compounds have shown therapeutic potential in disease models of cancer and as vaccine adjuvants. The therapeutic principle behind the use of these TLR agonists is to activate the immune system via interaction with the PRRs, so the dose of agonist used is critical. At too high a dose, therapeutic activity would be mitigated as too much interaction would lead to inflammation.

We also investigated the use of PS-ODNs and PS-ORNs containing modifications that neutralize the immune response as antagonists of TLR-7/9 and TLR-7/8/9 [92,93,94]. We showed that in animal models of autoimmune and inflammatory diseases, including psoriasis [95], lupus [96], Duchenne muscular dystrophy [96], colitis [97], and genetically defined lymphomas [98], such antagonists do indeed provide therapeutic benefit. Clinical benefit has also been shown in patients with psoriasis [99] and Waldenström’s macroglobulinemia [100]. In the case of antagonists, the therapeutic principle is blocking TLR interaction with endogenous PAMPs and thus avoiding their activation, but there is a danger that at too high a dose the antagonists could act as PAMPs themselves.

## 8. Connecting the Dots for New Antisense Chemistry

Armed with our much-improved understanding of antisense chemistry and potential interactions with PRRs, we wanted to further improve antisense functionality. We have already discussed the importance of 5′- end accessibility, so here we linked two molecules of the same antisense ODN together via their 5′ or 3′ ends to see how this would influence antisense potency and mechanism of action. To our surprise, we found that the 5′-5′ linked construct showed increased potency over the 3′-3′ and 3′-5′ linked PS-ODN dimers as well as control PS-ODN and gapmer designs both in vitro and in vivo [101]. We had really expected the 3′-3′ linked PS-ODN antisense to do best, based on the idea that such linking would provide increased nuclease stability as well as leaving the 5′ end accessible. In an attempt to understand the increased potency of the 5′-5′ linked construct, we investigated RNase-H mediated excision products by RACE-assay. When using the 5′-5′ linked PS- ODN, RNase-H cut the target RNA hybrids exclusively in the region of 8–11 nucleotides, whereas in all other antisense designs RNase-H cut towards the 5′ region of the antisense-RNA duplex. 

After more than two decades of intensive structure–activity relationship studies we now understand that the optimal design of antisense ODNs must maintain the accessibility of the 3′- end and use specific modifications in the 5′- end to modulate protein binding including PRRs (Figure 2).

## 9. Distractions and Side Roads along the Way

The story I have told here appears to be straightforward with a few difficulties that were easily solved and fortuitous results that only widened the therapeutic possibilities of oligonucleotides. However, to avoid giving the wrong impression, I would like to briefly mention some of the side roads and non-productive dead ends we encountered along the way. For example, we spent a huge amount of time trying to mask the polyanionic characteristics of PS-ODN using chemical or structural modifications. We tested a number of different approaches, from creating an ODN pro-drug [102] to using different structural designs (hairpins to lock the 3′ end [103], pseudocyclic structures [104], and using a pair of much shorter PS-ODNs targeting sequences next to each other [105]). None of these designs significantly increased therapeutic potency and looking back now we realize that our focus was on the 3′- end rather than the 5′- end, as it should have been [106]. As an aside, the morpholino (PMOs) chemistry was developed for the same reason—to eliminate ionization of the oligonucleotide in the usual physiological pH range [107]. In that chemistry, nucleic acid bases are bound to methylenemorpholine rings and linked through uncharged phosphorodiamidate groups instead of anionic phosphates. After many years of ups and downs, PMO antisense have shown encouraging results as splice modulators and three drugs have been approved.

We also extensively tested stereopure PS-ODNs. Because of the chiral nature of PS, ODNs containing this modification are a mixture of both Rp and Sp isomers at each PS modification (Figure 1H,I). For example, a 20-mer PS-ODN antisense with 19 PS linkages contains 2^19^ different stereoisomers. After optimizing the chemistry required for production of chirally defined oligonucleotides [108,109,110], we synthesized PS-ODNs that had stereopure linkages at defined positions, for example Rp-Sp-Rp or Sp-Rp-Sp. We learned a lot about these stereoisomers—affinity to target RNA was in the order Rp > Sp-Rp-Sp = Rp-Sp-Rp > mixture > Sp, nuclease stability was in the order Sp > Sp-Rp-Sp > mixture > Rp, and RNase-H activation was in the order Rp > mixture > Rp-Sp-Rp > Sp > Sp-Rp-Sp. However, increases in therapeutic potency of such chirally defined PS-ODNs compared to the random parental mixtures were small at best and not enough to justify the added complexity.

## 10. Summary

To close out this article, I would like to share a little bit of the wonder I feel about how far the field of oligonucleotide therapeutics has come during the last 30 or so years. When I started working with Paul, we had a grand vision of making oligonucleotides into therapeutics, but little idea about the long road that we would need to travel to get us there.

Now, over 70 antisense drug candidates employing gapmer chemistry or using steric blocking to modulate splicing have advanced to clinical trials, and to date seven drugs (gapmers mipomersen, inotersen, volanesorsen and splice modulating eteplirsen, nusinersen, golodirsen, and viltolarsen) have been approved by various regulatory agencies [111]. In addition, antisense is now being used for precision medicine, as demonstrated by the development of Milasen for the treatment of a child with a very particular mutation within one year [112]. As with all drug development, along the way many of the candidates have failed in late-stage clinical development, most recently tominersen for the treatment of Huntington’s Disease. It may be that the gene targets for these failed antisense ODNs were not optimal, that the levels of knockdown needed for therapeutic effect were not achieved, or that safety signals limited continuing dosing or dose escalation. But the answer for why certain antisense candidates have narrower therapeutic indexes than others may also lie in their particular sequence and how it interacts with the innate immune system. Simply put, the sequence and chemical modifications selected are essential features of RNA therapeutics.

I am proud that I was able to contribute to making Paul’s dream a reality, sadly too late for him to see it.
